# Thirty-minute screening of antibiotic resistance genes in bacterial isolates with minimal sample preparation in static self-dispensing 64 and 384 assay cards

**DOI:** 10.1007/s00253-015-6774-z

**Published:** 2015-07-31

**Authors:** Tanja Kostić, Michael Ellis, Maggie R. Williams, Tiffany M. Stedtfeld, John B. Kaneene, Robert D. Stedtfeld, Syed A. Hashsham

**Affiliations:** AIT Austrian Institute of Technology GmbH, Bioresources Unit, Konrad Lorenz Strasse 24, A-3430 Tulln an der Donau, Austria; Civil and Environmental Engineering, Michigan State University, East Lansing, MI 48824 USA; Center for Comparative Epidemiology, College of Veterinary Medicine, Michigan State University, East Lansing, MI 48824 USA; Center for Microbial Ecology, Michigan State University, East Lansing, MI 48824 USA; Barr Engineering Company, Ann Arbor, MI 48108 USA

**Keywords:** Antibiotic resistance, Rapid genetic testing, LAMP, Point of care, Gene-Z, *S. aureus*, *E. faecalis*, *E. faecium*

## Abstract

In a clinical setting, molecular assays such as polymerase chain reaction offer a rapid means to infer or confirm identity and therapeutic decisions. Accordingly, a number of molecular assays targeting identity and antibiotic resistance (AR) genes have been developed; however, these methods can be technically complex and relatively expensive. Herein, we describe a diagnostic concept utilizing isothermal amplification technology with non-purified heat-lysed cells and self-dispensing cards for testing multiple primers in parallel. This proof-of-concept study, performed with *Staphylococcus aureus* isolates and associated AR genes, was compared with culture-based susceptibility and quantitative PCR (qPCR). Results demonstrate reduced sample processing steps resulting in a turnaround time (starting from bacterial culture to ending in the antibiotic resistance gene profile) in less than 30 min. For antibiotics tested in which an associated AR gene was targeted on the Gene-Z card, 69 % (18/26) of culture-based resistance events were positive for related AR genes. A comparison of loop-mediated isothermal amplification (LAMP) and qPCR assays targeting the same antibiotic resistance genes showed a 98.2 % agreement in terms of presence and absence calls. Identity-based discrepancies between conventional (phenotypic) and molecular (genotypic) results were further resolved, and we were able to demonstrate higher accuracy in identification with the molecular analysis.

**Electronic supplementary material:**

The online version of this article (doi:10.1007/s00253-015-6774-z) contains supplementary material, which is available to authorized users.

## Introduction

Antibiotic resistance is a major public health problem worldwide (ECDC and EMEA 2009; Freire-Moran et al. 2011; French 2010; Gootz 2010; Levy and Marshall 2004; So et al. 2010; Spellberg et al. 2013). Infections caused by multidrug-resistant (MDR) organisms result annually in an estimated 25,000 deaths in Europe (29 countries) and 12,000 deaths in the USA (TATFAR 2011). The situation in developing countries is even worse (Byarugaba 2004; Okeke et al. 2005); for example, in 2010, the World Health Organization (WHO) estimated that 650,000 out of 12 million cases of tuberculosis were MDR-TB strains, in which case only slightly more than 50 % of patients with MDR-TB are expected to be cured (Chan 2012).

The gap between the burden of infections due to multidrug-resistant bacteria and the development of new antibiotics is well documented (ECDC and EMEA 2009; So et al. 2010; TATFAR 2011). It is also recognized that the development of new drugs alone will not be sufficient to address the growing resistance problem. Accordingly, CDC proposed four core actions to reduce spread of antibiotic resistances. These include the prevention and containment of infections, tracking resistant bacteria, improve the use of antibiotics, and the development of new antibiotics and new diagnostic tests for resistant bacteria (CDC 2013).

Conventional antibiotic resistance (AR) detection methods, e.g., broth dilution test, disk diffusion test, and automated instrument systems (Jorgensen and Ferraro 2009), are based on phenotypic characterization of isolated bacteria (susceptibility testing) and thus require extended turnaround times (Doern and Brecher 2011; Ledeboer and Hodinka 2011). Accordingly, molecular-based methods may offer a promising means to confirm identity and therapeutics, even if principally limited to detection of resistance determinants rather than susceptibility (Louie and Cockerill 2001). The US Food and Drug Administration (FDA) has approved several PCR-based tests for AR detection, including MRSA, vancomycin-resistant *Enterococcus* spp., and rifampin-resistant *Mycobacterium tuberculosis* (Ledeboer and Hodinka 2011). Nevertheless, it has to be considered that numerous AR mechanisms have been identified, and the number of involved genes is accordingly high (Giedraitienė et al. 2011; Liu and Pop 2009). Therefore, a comprehensive detection method should allow for significant multiplexing.

Accordingly, highly parallel microarray-based systems are a possible solution. One of the first microarrays for the detection of AR genes was developed over 10 years ago (Call et al. 2003) and targeted 18 AR genes. Successively, more comprehensive systems were developed (Antwerpen et al. 2007; Batchelor et al. 2008; Card et al. 2013; Dally et al. 2013; Frye et al. 2006, 2010; Fu et al. 2012; Garneau et al. 2010; McNicholas et al. 2011; Monecke and Ehricht 2005; Monecke et al. 2012; Perreten et al. 2005; Strommenger et al. 2007; van Hoek et al. 2005; Weile et al. 2007; Zhu et al. 2007). However, none of these techniques are routinely used. Probable explanation lies in the highly technical complexity of methods and analysis. Standard microarray protocols include DNA extraction, DNA amplification and labeling, hybridization, washing, scanning, and data analysis. These steps are time consuming (at least several hours) and not easily automated. Furthermore, the potential for error increases with each manual step. Therefore, simpler and more rapid solutions are required for adoption and routine use.

Gene-Z, a novel device for the point-of-care genetic testing, combines multiplexing potential of the microarray (Stedtfeld et al. 2012; Tourlousse et al. 2012) with simplicity of loop-mediated isothermal amplification (LAMP). LAMP is an established nucleic acid isothermal amplification method offering rapid, accurate, and cost-effective detection (Mori and Notomi 2009). LAMP utilizes four to six primers targeting six to eight regions on the target gene, and two of the primers (termed loop) are optionally used to reduce amplification time from 60–90 min to less than 30 min (Nagamine et al. 2002). Strand displacement activity of *Bst* polymerase and single-stranded loops generated by primer structure allow amplification without temperature cycling. High amplicon yield of LAMP permits detection with simple optics or the naked eye (Tomita et al. 2008; Soli et al. 2013). Furthermore, LAMP is more robust in terms of input material and does not have sample preparation requirements compared to PCR (Dugan et al. 2012).

In this study, we investigated the potential of using the disposable self-dispensing cards developed for the Gene-Z system for identification and profiling AR genes from bacterial isolates with an emphasis on simplification of sample preparation and time reduction. In detail, novel LAMP assays targeting AR genes were initially tested with both reference strains and 30 bacterial isolates using a conventional real-time thermal cycler. The selected assays were subsequently screened with a second set of 11 bacterial isolates using 64-well and 384-well disposable Gene-Z cards. Efficiency of LAMP reactions was also tested with genomic DNA (gDNA), cells, and crude lysates from the bacterial isolates. Presence/absence calls of AR genes, determined via visual inspection of time lapse images captured in real time on the Gene-Z card, were compared to phenotypic identification methods and susceptibility. Gene-Z card results were also compared with LAMP and qPCR run in vials using a conventional real-time cycler.

## Materials and methods

### AR gene selection and LAMP primer design

Antibiotic Resistance Genes Database (ARDB, Liu and Pop 2009) was used to assemble a list of AR genes present in *Enterococcus faecalis*, *Enterococcus faecium*, and *Staphylococcus aureus* as of June 2010. For the proof-of-concept study, gene selection was based on the following criteria: (i) the number of journal articles found in PubMed when searching for the gene name, (ii) the number of sequences listed in ARDB (Liu and Pop 2009), (iii) the number of strains in which the gene had been observed, (iv) coverage of a wide range of AR gene categories, and (v) the ability to design LAMP primers from the gene.

The web tool Primer Explorer (http://primerexplorer.jp/e/) was used for LAMP primer design. One representative sequence was initially used for primer design. Specificity of designed primer sets was confirmed by BLAST analysis (http://blast.ncbi.nlm.nih.gov/). For some primer sets, degenerate bases were used to increase coverage. It should be noted that primers were designed to be gene specific, not species specific. As such, some primer sets target AR genes present in species other than *E. faecalis*, *E. faecium*, and *S*. *aureus*.

### Reference strains and bacterial isolates

Assay was initially tested using gDNA of bacterial reference strains (Table 1) obtained from the American Type Culture Collection (ATCC). Screening isolates were collected by The Center for Comparative Epidemiology at the MSU College of Veterinary Medicine. The first screening group consisted of 30 isolates (10 isolates of each *E. faecalis*, *E. faecium*, and *S. aureus*). A second screening group, which was also subjected to culture-based susceptibility testing, consisted of 11 *S. aureus* isolates (Table 2).

**Table 1:** Antibiotic resistance genes selected for LAMP primer design

Gene	Resistance mechanism [gene ontology #]	Antibiotic	Target organism(s)	Reference Strain used for validation (ATCC #)	# of genera^a^
*ant3ia*	Aminoglycoside nucleotidyltransferase activity [GO:0034068]	Spectinomycin, streptomycin	*E. faecalis*	*A. baumannii* (BAA-1710)	30
*aadD*	Aminoglycoside nucleotidyltransferase activity [GO:0034068]	Kanamycin, tobramycin	*S. aureus*	*S. aureus* Mu50 (700699)	3
*bacA*	Di-trans,poly-cis-decaprenylcistransferase activity [GO:0008834]	Bacitracin	*E. faecalis*, *S. aureus*	*S. aureus* Mu50 (700699)	153
*bl2B*_*tem*	Beta-lactamase activity [GO:0008800]	Cephalosporin, penicillin	*S. aureus*	*E. fergusonii* (35469)	34
*ble*	Binding protein	Bleomycin	*S. aureus*	*S. aureus* Mu50 (700699)	5
*cata9*	Chloramphenicol *O*-acetyltransferase activity [GO:0008811]	chloramphenicol	*E. faecium*, *S. aureus*	*S. pneumoniae* (700669)	5
*dfra12*	Dihydrofolate reductase activity [GO:0004146]	Trimethoprim	*E. faecalis*, *S. aureus*	–	12
*lsa*	ABC efflux family that is resistant to MLS antibiotics	Lincosamide, macrolide, streptogramin B	*E. faecalis*	*E. faecalis* V583 (700802)	1
*mphC*	Transferase activity, transferring phosphorus-containing groups [GO:0016772]	Macrolide	*S. aureus*	–	2
*mepA*	Multidrug efflux pump activity [GO:0015559]	Tigecycline	*S. aureus*	*S. aureus* Mu50 (700699)	1
*norA*	Permease	Fluoroquinolone	*S. aureus*	*S. aureus* Mu50 (700699)	1
*qacA*	MULTIDRUG efflux pump	qa-compound	*S. aureus*	*S. aureus* Mu50 (700699)	1
*tetO*	Translation elongation factor activity [GO:0003746]	Tetracycline	*E. faecalis*	*S. pyogenes* (12344)	16
*tetM*	Translation elongation factor activity [GO:0003746]	Tetracycline	All	*S. aureus* Mu50 (700699)	33
*vanG*	d-Alanine-d-alanine ligase activity [GO:0008716]	Vancomycin	*E. faecalis*	–	1
*vanB*	d-Alanine-d-alanine ligase activity [GO:0008716]	Vancomycin	*E. faecalis*, *E. faecium*	*S. aureus* Mu50 (700699)	5
*vanA*	d-Alanine-d-alanine ligase activity [GO:0008716]	Teicoplanin, vancomycin	All	–	5
*vanYA*	d-alanine-d-alanine ligase activity [GO:0008716]	Teicoplanin, vancomycin	All	–	4
*vatA*	Acetyltransferase activity [GO:0016407]	Streptogramin A	*S. aureus*	–	1
*vgbA*	Lyase activity [GO:0016829]	Streptogramin B	*S. aureus*	–	1

^a^Total number of genera that harbor the selected antibiotic resistance gene, as listed by ARDB (Liu and Pop 2009)

**Table 2:** Culture-based antibiotic resistance and presence (+) and absence (−) of AR gene elements observed with LAMP assays tested on Gene-Z cards with crude heat-lysed non-purified cell templates

Isolate number	Phenotypic antibiotic resistance (MIC-μg/mL)	LAMP assay (*T*_*t*_, min)				
		*norA*	*ble*	*tetM*	*mecA*	*nuc*
*S. aureus* Mu50	Not tested	+(27)	+(21)	+(18)	+(22)	+(20)
AR131	Amp (1), Pen (4), Rif (>4), Cli (>2), Ery (>4), Oxa (>8), Syn (>8), Tet (>16)	−	−	+(21)	−	−
AR132	Amp (8), Pen (>8), Cip (>2), Ery (>4), Gat (4), Lev (8), Oxa (>8)	+(30)	−	−	+(24)	+(24)
AR133	Pen (0.5), Cip (>2), Cli (>2), Ery (>4), Tet (>16)	−	−	+(25)	−	−
AR134	Amp (>16), Pen (>8), Cef (>64), Cip (>2), Cli (>2), Ery (>4), Gat (8), Lev (>8), Oxa (>8)	+(26)	+(17)	−	+(18)	+(22)
AR135	Amp (8), Pen (>8), Cef (>64), Cip (>2), Ery (>4), Gat (4), Lev (>8), Oxa (>8)	+(30)	+(19)	−	+(18)	+(21)
AR136	Amp (>16), Pen (>8), Cef (>64), Cip (>2), Cli (>2), Ery (>4), Gat (>8), Lev (>8), Oxa (>8)	+(29)	+(18)	−	+(18)	+(22)
AR137	Rif (>4), Cip (>2), Ery (>4), Gat (>8), Lev (>8), Syn (>4), Tet (>16), Tri (>4), Van (>128)	+(35)	−	−^a^	−	+(22^a^)
AR139	Amp (>16), Pen (>8), Cef (>64), Cli (>2), Ery (>4), Gen (16), Oxa (>8)	−	−	−	+(26)	−
AR141	Amp (>16), Pen (>8), Cef (>64), Cip (>2), Cli (>2), Ery (>4), Gat (>8), Lev (>8), Oxa (>8)	+(31)	+(16)	−^a^	+(22)	+(21)
AR142	Amp (>16), Pen (>8), Cip (>2), Ery (>4), Gat (8), Lev (>8), Oxa (>8)	+(41)	+(31)	−	+(26)	+(28)
AR143	Amp (>16), Pen (>8), Cef (>64), Cip (>2), Cli (>2), Ery (>4), Gat (8), Lev (>8), Oxa (>8)	+(35)	+(31)	−^a^	+(24)	+(20)

Mean threshold time (*T*_*t*_, min) for three replicate reactions is listed in parentheses

*Amp* ampicillin, *Pen* penicillin, *Rif* rifampin, *Cli* clindamycin, *Ery* erythromycin, *Oxa* oxacillin, *Syn* quinupristin-dalfopristin, *Tet* tetracycline, *Cip* ciprofloxacin, *Gat* gatifloxacin, *Lev* levofloxacin, *Cef* ceftriaxone, *Tri* trimethoprim-sulfamethoxazole, *Van* vancomycin, *Gen* gentamicin

^a^Ambiguous results with endpoint image analysis; thus, images captured in real time were used for calls of presence and absence

A microdilution system (Sensitire microdilution system, TREK Diagnostic Systems Inc., Cleveland, OH, USA) was used to perform antibiotic susceptibility testing (for a second screening group) on a commercially prepared plate (GPN3F Gram-positive MIC plate with tigecycline, TREK Diagnostic Systems Inc., Cleveland, OH, USA). Ampicillin, oxacillin, penicillin, ceftriaxone, ciprofloxacin, levofloxacin, gatifloxacin, clindamycin, daptomycin, erythromycin, gentamicin, vancomycin, linezolid, quinupristin-dalfopristin, rifampin, tetracycline, and trimethoprim-sulfamethoxazole were used in susceptibility testing. Reference strains *E. faecalis* (ATCC 29212) and *S. aureus* (ATCC 29213) were used for quality control purposes. Quality control results were reviewed for each batch of tests, all of which were within acceptable limits. Inducible clindamycin resistance was not investigated. A fluorescence technology-based automated reading system (AutoReader, TREK Diagnostic Systems Inc., Cleveland, OH, USA) was used to generate antibiotic susceptibility and resistance profiles (Table S2). Susceptibility, intermediate resistance, and resistance were determined by comparison with Clinical and Laboratory Standards Institute breakpoints (Clinical and Laboratory Standards Institute 2007).

### Template preparation

All strains were grown in trypticase soy broth (TSB) (211768, BD Diagnostic Systems, Sparks, MD, USA) over night at 37 °C. Determination of colony forming units (CFU) was done by drop-plating (Herigstad et al. 2001) ten-fold dilution series in triplicate onto trypticase soy agar (TSA) (211042, BD, Diagnostic Systems, Sparks, MD, USA).

Initial testing of primers sets with reference strains and the first screening with isolates were performed with gDNA templates. Genomic DNA was extracted using QIAGEN DNeasy Blood and Tissue Kit (69504, QIAGEN, Valencia, CA, USA) following the protocol for Gram-positive bacteria. DNA concentration was measured using QUBIT dsDNA BR Assay (Q32850, Life Technologies, Grand Island, NY, USA) and adjusted to 1 ng/μL.

The second screening of isolates was performed using crude lysates. For this purpose, one colony from TSA plate was re-suspended in 200 μL 1× PBS (P5493-1L, Sigma Aldrich, St. Louis, MO, USA) and heat lysed at 95 °C for 5 min.

### LAMP amplification

LAMP reactions were performed as described previously (Stedtfeld et al. 2012). Briefly, reaction mixtures contained 1× *Bst* DNA polymerase buffer (M0275L, New England Biolabs, Ipswich, MA, USA), 1.4 mM of each dNTP (10297018, Life Technologies, Grand Island, NY, USA), 800 mM betaine (B0300, Sigma Aldrich, St. Louis, MO, USA), 6 mM MgSO_4_ (B1003S, New England Biolabs, Ipswich, MA, USA), 1× primer mix (1.6 μM FIP and BIP, 800 nM LF and LB and 200 nM F3 and B3 primers), 1 mg/mL bovine serum albumin (B9000S, New England Biolabs, Ipswich, MA, USA), 20 μM SYTO-81 (S11362, Molecular Probes, Eugene, OR, USA [product not available anymore, can be replaced with SYTO-82, S11363, Life Technologies, Grand Island, NY, USA]), 0.2 % Pluronic F-68 (24040032, Life Technologies, Grand Island, NY, USA), and 0.64 U/μL *Bst* DNA polymerase (large fragment) (M0275L, New England Biolabs, Ipswich, MA, USA). Reactions were performed with 1 ng gDNA or 1 μL of cell lysate in 10 μL LAMP reaction.

Reactions were tested using a conventional real-time cycler (Chromo4, BioRad Laboratories, Hercules, CA, USA) under isotheral conditions or on Gene-Z cards. For on-card reactions, 1 μL/well (64-well card) or 0.25 μL/well (384-well card), 1× primer mix was dispensed and dehydrated directly into card wells prior to assembly. For a 64-well card, 60-μL reaction mixtures (including 1 μL template per 10 μL reaction mix) were prepared and loaded per lane (16 reaction wells). For 384-well card, 150-μL reaction mixtures (including 1 μL template per 10 μL reaction mix) were prepared and loaded into one of two inlets (192 reaction wells each).

All reactions were performed at 63 °C for 1 h with real-time imaging; every 45 s in the real-time Chromo4 cycler and every 60 s on card using a CCD camera as described previously (Ahmad et al. 2011; Tourlousse et al. 2012). Briefly, cards were incubated on a digital heater (Labnet International Inc., Edison, NJ, USA). A 530-nm green LED (05027-PM12, LED Supply, Randolph, VT, USA) was used to excite fluorescence, and a 0.25-megapixel monochrome CCD camera (MEADE DSI Pro, Irvine, CA, USA) with a 572 ± 20 nm bandpass filter (FF01-572/2825, Semrock, Rochester, NY, USA) was used to capture fluorescent emission.

### Card fabrication

**Fig. 1:**
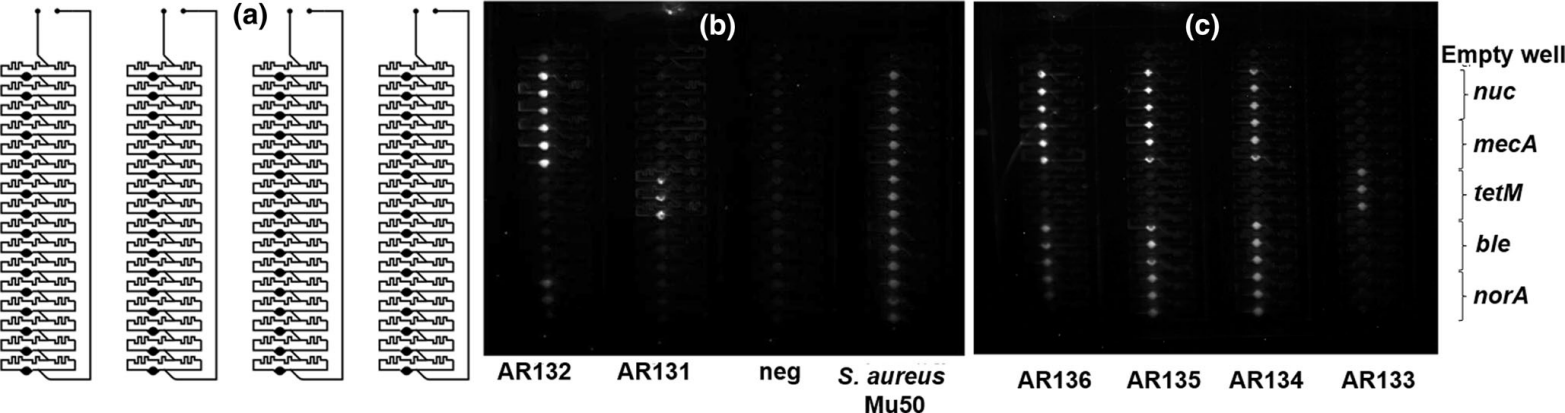
Testing 64-well card with crude cell lysates of *S. aureus* isolates. **a** Schematic diagram of 64-well Gene-Z card (four lane version for four samples). **b**–**c** Fluorescence images of Gene-Z card after 60 min amplification reactions for two of the tested cards. The layout of primers dehydrated in the card during assembly and the identity of the samples added in each array are indicated

**Fig. 2:**
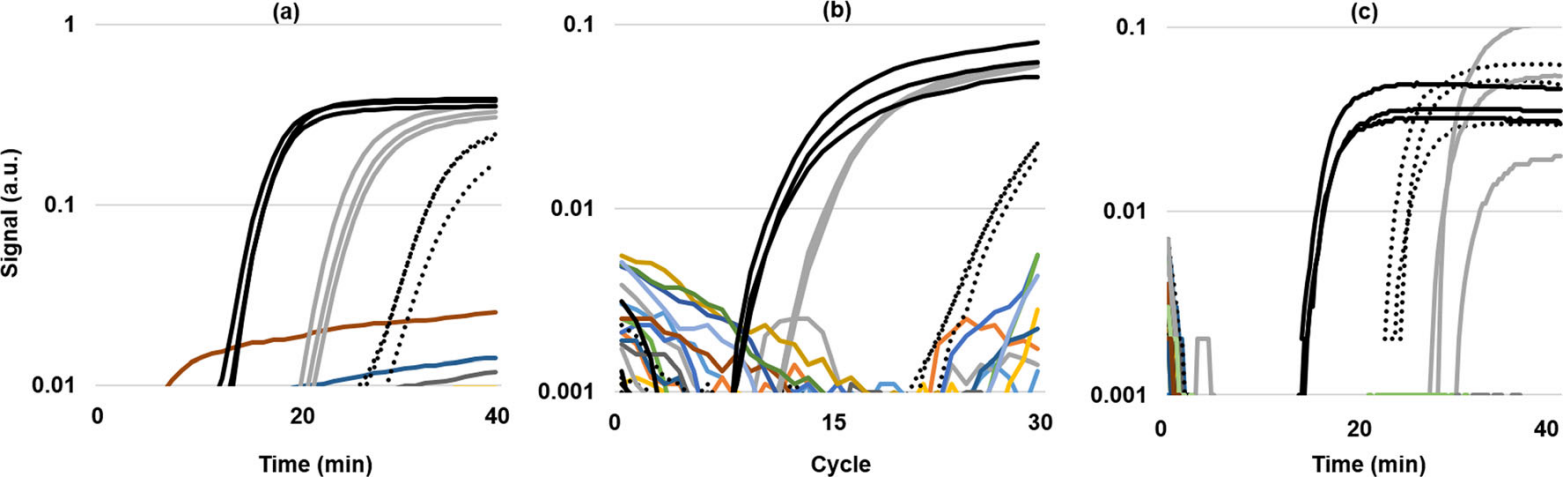
Real-time amplification curves for AR139 tested via **a** LAMP in vials with heat-lysed non-purified cell template, **b** qPCR in vials with gDNA template, and **c** LAMP on Gene-Z cards with heat-lysed non-purified cell template. *Solid black curves*, *gray curves*, and *dotted black curves* are assays targeting the 16S rRNA gene specific to *S. aureus*, *qacA* gene, and *mphC* gene

Cards were designed to allow for dispensing of sample into a series of reaction wells without carryover of predispensed primers (Stedtfeld and coauthors, submitted to Biomedical Microdevices). Briefly, channels and wells were cut into 1.6-mm polymethyl methacrylate (PMMA) sheets (8560K173, Mcmaster-Carr, Chicago, IL, USA) using a commercially available desktop CO_2_ laser (MLE-40, Full Spectrum Laser LLC, Las Vegas, NV, USA). The cutting power and speed of the laser were varied to obtain desired depth and thickness of channels and wells. Both 64-well and 384 well cards were fabricated and tested for this study. For this study (Figs. 1a and 2a), 64-well cards were fabricated with four separate lanes, each with 16 wells, and 384-well cards were designed with two lanes each with 196 wells. The volume of wells in the 384-well cards was 0.4 μL. The sample volume for the 64-well card (all four inlets) was 240 μL (i.e., 60 μL per inlet) and for the 384-well card (including both inlets) was 300 μL.

After laser micromachining, the card was cleaned with 70 % ethanol to remove PMMA residue and dried with filtered compressed air to remove dust and other airborne particles. For cards tested with LAMP, primers were dispensed into wells and dehydrated by placing the card on a bench-top heater at 70 °C for approximately 10 min. Subsequently, engraved channels and wells were enclosed via a biocompatible optical film with pressure-sensitive adhesive (4311971, MicroAmp Optical Adhesive Film; Applied Biosystems, Carlsbad, CA, USA). Secure bonding of the film to PMMA was ensured using a press (4386; Carver, Wabash, IN, USA) at 3000 lb of pressure.

The card was loaded by first using a needle to pierce the film above the loading port, followed by adding a sample using a conventional 200-μL pipette. Pressure exerted by the pipette pushed sample into the channels and reaction wells. In this process, air inside the micro-channels was purged out through a single air vent placed downstream from the series of reaction wells. Fast-drying epoxy (SY-QS, Super Glue Corp., Rancho Cucamonga, CA, USA) was used to seal inlet and outlet (air) vents.

### Gene-Z card versus qPCR assays

To compare Gene-Z results with more traditional molecular methods, qPCR assays were tested with four of the isolates (AR131, AR135, AR139, AR142) and *S. aureus* strain Mu50 using vials in a conventional real-time cycler (Chromo4, BioRad Laboratories). Genes targeted for this comparison included *ant3ia*, *aadA*, *dfra12*, *mphC*, *qacA*, *tetM*, and *mecA* genes, in which qPCR primers were previously described (Looft et al. 2012; Zhu et al. 2013). In addition, the F3 and B3 LAMP primers were used as forward and reverse qPCR primers for the *norA*, *ble*, *nuc*, and 16S ribosomal RNA (rRNA) gene specific to *S. aureus.* qPCR was performed in 25-μL volumes and consisted of 500 nM forward and reverse primers, 10 ng gDNA, and reagents from the Power SYBR Green PCR Master Mix (Life Technologies). Real-time reactions were run using the Chromo4 and included a 10-min enzyme activation at 95 °C followed by 40 cycles of 95 °C for 15 s and 60 °C for 60 s. A no-template control was included for all assays, and all reactions were run in triplicate vials.

LAMP reactions were also tested in vials using the Chromo4. LAMP reactions consisted of 0.4 μL per reaction cell lysate on the Gene-Z card and 1 μL cell lysate per reaction in vials on the Chromo4. For the Gene-Z card, primers were dispensed in triplicate wells, and two lanes (each with 16 wells) were used per isolate. With this configuration, two isolates were tested against ten assays with each Gene-Z card. Gene-Z cards were monitored in real time using the Gene-Z handheld device (Stedtfeld et al. 2012). Fluorescence of wells was captured in 16-s intervals with the Gene-Z device and in 1-min intervals in the cycler. Temperature was maintained at 63 °C in both platforms.

### Data analysis

Endpoint image analysis of Gene-Z cards consisted of obtaining the signal intensities of the second (2 min) and last image (60 min), which were extracted from 16-bit images using ImageJ software package (http://rsbweb.nih.gov/ij/). Absolute (Δ*R*_*n*_ = *S*_60_ − *S*_2_) and relative (percent change = *S*_60_/*S*_2_ * 100) signal changes were calculated using Microsoft® Office Excel. Given the large differences in the background intensity (*S*_2_ = 2–200), percent change was considered to be a more reliable amplification indicator than Δ*R*_*n*_. The large difference in background intensity caused some ambiguity in making presence and absence calls based solely on initial and endpoint image analysis. In these cases, time lapse images taken during the amplification reaction was used to confirm presence or absence and to determine threshold time (*T*_*t*_). An example of this ambiguity is demonstrated in Fig S1. Overall, results were considered positive if 2 out of 3 wells exhibited percent change value greater than 115 % (i.e., signal increase of minimal 15 %). This cutoff value is arbitrarily selected based on high background of initial images.

For real-time analysis, qPCR threshold cycle (*C*_*t*_) was calculated as time in which the signal increased 0.01 (arbitrary units) above the original signal. For real-time analysis of LAMP in Gene-Z cards and in vials, the threshold time (*T*_*t*_) was calculated as the time in which the signal increased 0.01 and 0.03 (arbitrary units), respectively, above the original signal.

### 16S rRNA gene PCR and sequencing

For ambiguities between phylogenetic and molecular assays, further clarification was achieved via sequencing PCR amplicons targeting the 16S rRNA gene. The 16S rRNA gene was amplified using primers Fu16 (5′ CCTACGGGAGGCAGCAG 3′) and Ru16 (5′ GACGTCRTCCNCDCCTTCCTC 3′) following previously published protocols ( Khalaj-Kondori et al. 2007). Genomic DNA was isolated with QIAGEN DNeasy Blood and Tissue Kit (69504, QIAGEN, Valencia, CA, USA) following the protocol for Gram-positive bacteria. Amplicons were purified using the QIAquick PCR purification Kit (28104, QIAGEN, Valencia, CA, USA), and sequencing was performed by the Research Technology Support Facility (RTSF) at Michigan State University using the ABI 3730xl platform (Applied Biosystems, Grand Island, NY, USA).

Based on the 16S rRNA gene amplicons, isolate AR 139 had 100 % sequence identity to *Staphylococcus* spp. (NCBI accession number KF575164.1), isolate AR133 had 100 % sequence similarity to *Staphylococcus* spp. (NCBI accession number NR_102784.1), and isolate AR131 had 99.7 % sequence similarity to *E. faecalis* (NCBI accession number KF193427.1). The partial 16S rRNA gene sequence from AR131 was submitted to NCBI (KP824986).

## Results

### Design of AR gene LAMP assays

In total, 96 AR genes were identified in *E. faecalis*, *E. faecium*, and *S*. *aureus* (Table S3). Some genes were exclusive to one organism of interest, whereas other genes were present in two or three of the target organisms. Some genes are also not specific to the three organisms examined in this study. Twenty candidate target genes were selected for LAMP primer design based on clinical and scientific relevance (Table 1, Table S1). In two cases (*cata9* and *qacA*), loop primer F (LF primer) could not be designed.

Thirteen out of 20 LAMP assays were initially tested using gDNA from ATCC (Table 1), and reactions were performed in the Chromo4. Detection threshold times (*T*_*t*_) ranged from 10 to 20 min using a 1-ng gDNA template (equivalent to 3.22 × 10^5^ genome copies of *S. aureus* Mu50). Agreement between expected and experimental results was observed in terms of specificity and discrepancies were clarified. For example, *S. aureus* Mu50 yielded positive results with *aadD*, *bacA*, *ble*, *mepA*, *norA*, *qacA*, and *tetM* primer sets. With the exception of *norA*, this is in agreement with the AR gene information on *S. aureus* Mu50 listed in the ARDB (Liu and Pop 2009). While the *norA* gene is not listed as one of the *S. aureus* Mu50 AR genes in the ARDB, cross-referencing with the complete genome sequence of *S. aureus* Mu50 (NCBI accession number BA000017) showed the *norA* gene is present in Mu50. Based on initial tests with reference strains, LAMP assays were used in isolate screening experiments.

### Minimal sample preparation

A comparative experiment was performed using gDNA, crude heat-lysed non-purified cells, and native cells of *S. aureus* isolate AR132 as template. LAMP amplification was performed using Chromo4. Results demonstrate LAMP can be performed with minimal sample preparation of *S. aureus* isolates. Crudely lysed cells had a lower *T*_*t*_ compared to native cells (Table S4, Fig. S2), and both crude samples (native and lysed) yielded lower *T*_*t*_ values compared to gDNA samples. This can be partially explained by a smaller amount of template in reactions tested with gDNA. In detail, plate counts of colony forming units (CFU) showed reactions tested with native cells had approximately 5 × 10^5^ CFU/μL. *S. aureus* has an average genome size of 2.8 Mbp (Suzuki et al. 2012); 1 ng gDNA corresponds to approximately 3.3 × 10^5^ genome copies. As such, amplification reactions performed with gDNA theoretically had a slightly lower target number. In addition, plate counts do not include extracellular DNA or non-viable cells.

### Screening first group of isolates

Initial screening was performed with 30 isolates to further test LAMP assays targeting AR genes and to aid in selection of genes to test with Gene-Z card. Experiments were performed using gDNA of *S. aureus*, *E. faecalis*, and *E. faecium* isolates (10 each) and LAMP reactions were performed under standard reaction conditions (1 ng gDNA template, 10 μL reaction volume, real-time detection in Chromo4). Out of the seven primer sets not initially tested with reference strains, four targeting the *mphC*, *vanA*, *vanYA*, and *vatA* genes yielded positive results with one or more of the isolates (Table S5). Multiple resistance events were more common in *S. aureus* isolates (10/10) than in *E. faecalis* (3/10) or *E. faecium* (5/10) isolates. No AR genes were detected in six of the tested isolates (two *E. faecalis* and four *E. faecium*), which may be a result of investigating only a fraction of the genes listed in the ARDB (20 out of 96).

### Second group of isolates in Gene-Z cards, qPCR, and susceptibility profiles

A second screening was performed with 11 *S. aureus* isolates to demonstrate utility of the Gene-Z card for testing multiple assays simultaneously and for comparing with culture-based susceptibility. Cards were fabricated with 64 reaction wells (Fig. 1a) and four individual loading ports and lanes. Gene-Z cards were tested with heat-lysed non-purified cell templates. With this configuration, four samples (each with 16 reaction wells) were tested per card. Five different primer sets were predispensed per sample lane in triplicate. Selected assays included *norA*, *ble*, and *tetM* and previously developed *mecA* and *nuc* assays (Table S1). The *nuc* gene, a thermostable nuclease of *S. aureus*, commonly used as a species-specific marker (Brakstad et al. 1992) was included to confirm identity. The *mecA* assay (beta-lactam/methicillin resistance) was included because of its importance in *S. aureus* antibiotic resistance (Stefani et al. 2012; Wielders et al. 2002). Brighter wells (e.g., all wells in the lane loaded with *S. aureus* Mu50) following the 60-min reaction are considered positive amplification events (Fig. 1b, c). Summarized results for all of the cards and isolates show a majority of the positive assays amplified after ~20–25 min (Table 2). The *norA* gene is the exception, amplifying around 30 min.

To compare Gene-Z card with more traditional molecular methods (i.e., qPCR), four of the isolates (AR131, AR135, AR139, and AR142) and *S. aureus* Mu50 were also tested using LAMP (heat-lysed non-purified cell template) and qPCR (gDNA) in vials run in a conventional real-time cycler (Fig. 2). Overall, 100 % agreement was observed between LAMP with Gene-Z card and LAMP in vials and 98.2 % between and qPCR in vials (Table 3). Disagreement was observed for the assay targeting the 16S rRNA genes specific to *S. aureus* when tested with AR131, which amplified with qPCR but not with LAMP. Sequencing of the 16S rRNA gene showed the AR131 isolate had 99.7 % sequence similarity to *E. faecalis*; thus, amplification with qPCR may have been a lack of primer specificity. Probe match results obtained using the Ribosomal Database Project (Cole et al. 2014) verified that the qPCR primer designed to target the 16S rRNA gene of *S. aureus* also targeted 77 sequences from *Enterococcus*.

**Table 3:** Presence and absence (−) of gene targeted assays observed with LAMP Gene-Z cards (crudely lysed cell template), LAMP in conventional vials (crudely lysed cell template), and qPCR assays in conventional vials (gDNA template)

Isolate/Target	*aadA*	*ble*	*mecA*	*mphC*	*norA*	*nuc*	*qacA*	*tetM*	16S *S. aureus*
AR131									
LAMP vial	−	−	−	−	−	−	−	19.3 ± 3.0	-
qPCR vial	−	−	−	−	−	−	−	15.3 ± 0.6	13.3 ± 0.6
LAMP Gene-Z	−	−	−	−	−	−	−	14.0 ± 0.4	−
AR135									
LAMP vial	26.6 ± 0.6	16.6 ± 0.6	15.3 ± 0.6	−	38.3 ± 0.6	17.0 ± 0.0	−	−	15.6 ± 0.6
qPCR vial	14.0 ± 0.0	15.6 ± 0.6	16.6 ± 0.6	−	36.6 + 0.6	15.6 + 0.6	−	−	12.3 ± 0.6
LAMP Gene-Z	16.4 ± 0.2	20.2 ± 0.7	18.2 ± 0.2	−	22.8 ± 0.2	14.8 ± 0.2	−	−	16.8 ± 1.2
AR139									
LAMP vial	−	−	15.6 ± 0.6	31.3 ± 0.6	−	−	23.6 ± 0.6	−	15.6 ± 0.6
qPCR vial	−	−	16.6 ± 0.6	28.0 ± 0.0	−	−	16.0 ± 0.0	−	12.6 ± 0.6
LAMP Gene-Z	−	−	26.3 ± 0.2	23.2 ± 1.1	−	−	28.3 ± 1.2	−	14.6 ± 0.4
AR142									
LAMP vial	26.3 ± 1.1	17.0 ± 1.0	15.3 ± 0.6	−	25.0 ± 0.0	17.3 ± 0.6	−	−	16.0 ± 0.0
qPCR vial	17.0 ± 0.0	16.3 ± 0.6	17.0 ± 0.0	−	19.3 ± 1.5	15.3 + 0.6	−	−	13.3 ± 0.6
LAMP Gene-Z	14.5 ± 0.2	18.2 ± 0.3	26.3 ± 0.2	−	24.0 ± 2.6	16.2 ± 0.2	−	−	16.1 ± 0.3
*S. aureus* Mu50									
LAMP vial	15.3 ± 0.6	12.3 ± 0.6	13.0 ± 0.0	−	19.6 ± 3.2	13.6 ± 0.6	19.0 ± 0.0	10.6 ± 0.6	13.0 ± 0.6
qPCR vial	15.3 ± 0.6	15.6 ± 0.6	19.3 ± 4.0	−	16.6 ± 0.6	16.3 ± 0.6	15.3 ± 0.6	15.6 ± 0.6	13.0 ± 0.0
LAMP Gene-Z	18.3 ± 1.1	17.9 ± 1.0	22.2 ± 0.6	−	17.8 ± 2.1	17.2 ± 1.8	27.0 ± 0.9	11.2 ± 0.6	11.8 ± 0.6

Mean threshold time (*T*_*t*_, min) for three replicate reactions is listed with standard deviation. Assays targeting the *ant3ia* and *dfra12* antibiotic resistant genes did not amplify in all cases and are not included

### Experiments with 384-well card

**Fig. 3:**
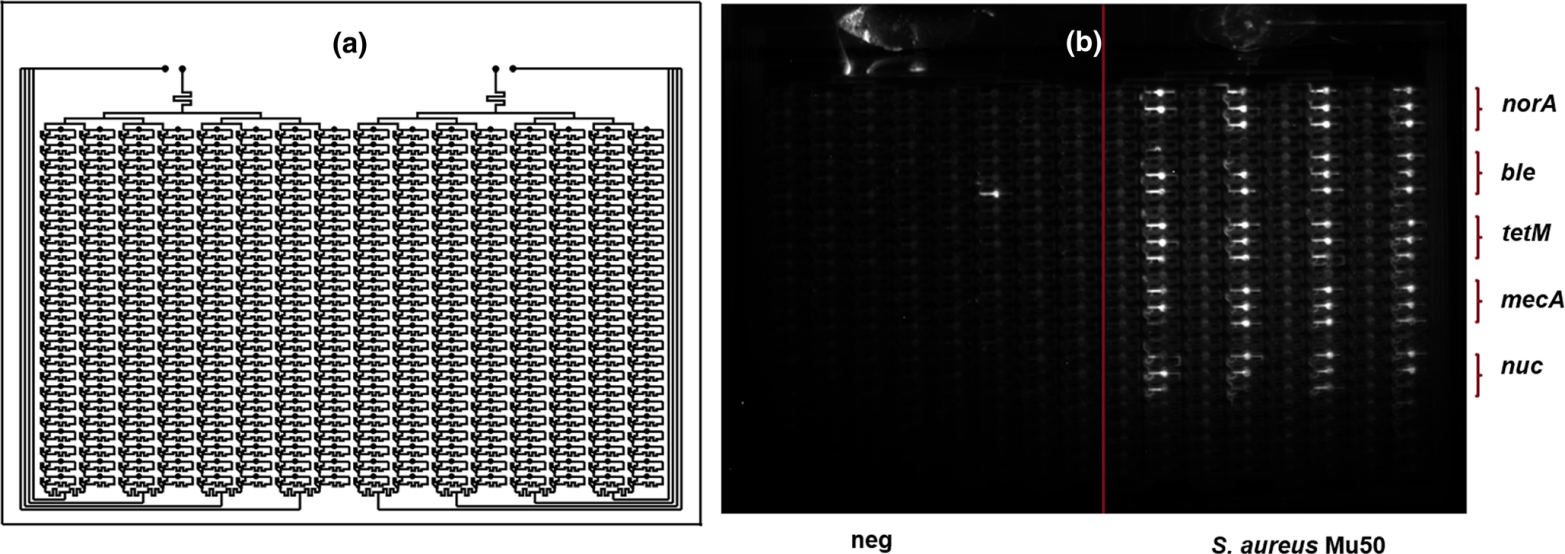
Testing 384-well card with gDNA of *S. aureus* Mu50. **a** Schematic diagram of 384-well card (two-inlet version for two samples). **b** Fluorescence image of 384-well card after 60 min amplification. The layout of primers dehydrated in the card during assembly and composition of the samples added in each array are indicated. The lone well with high signal in the no-template control may be due to primer dimers as only 1 of 12 replicate wells showed amplification

Considering results and 90 AR genes are present in these three organisms tested (Table S3), it is clear the 64-well card may not be sufficient for applications requiring more comprehensive screening. Therefore, a 384-well card with self-dispensing microfluidic channels was tested. Given the denser architecture and reduced reaction volume compared to the 64-well card, tests were performed to ensure no cross reactivity or carryover of primers between wells. For this, the same five primer sets used for the 64-well card were also used with the 384-well card. LAMP amplification was demonstrated with both gDNA template (Fig. 3b) and lysed cells of *S. aureus* Mu50. Positive/negative calls in the 384-well card correspond to results observed on the 64-well card. Amplification time was comparable to 64-well card, and no carryover of primer or amplified product was observed between wells. One false-positive signal (out of 192 wells) observed in a well loaded with no template control (Fig. 3b) may be due to primer dimers; however, this is speculation as the amplicon was not analyzed following the reaction.

## Discussion

LAMP assays screened with the 64-well Gene-Z card correctly identified organisms originally misclassified via culturing. In detail, three isolates (AR131, AR133, and AR139) previously identified as *S. aureus* tested negative for the LAMP assay targeting the *nuc* gene. The *nuc* gene is a *S. aureus*-specific marker (Brakstad et al. 1992), and accordingly, a positive result was expected for all isolates. The three isolates were further tested using *Staphylococcus* spp.-specific 16S LAMP assay (Table S1), and positive signals were only observed with AR133 and AR139. To further clarify discrepancies, a partial 16S rRNA gene (675-bp fragment) was PCR amplified and sequenced. Sequence analysis revealed isolate AR131 is most related to *E. faecalis* (99.7 % sequence homology to KF193427.1, new accession number submitted KP824986). Isolates AR133 and AR139 are more related to *Staphylococcus intermedius*/*Staphylococcus pseudintermedius* (100 % sequence homology to NR_102784.1) and *Staphylococcus haemolyticus*/*Staphylococcus epidermidis* (100 % sequence homology to KF575164.1), respectively. As such, the three isolates are not *S. aureus* confirming the absence of the *nuc* gene as observed with the LAMP assay.

LAMP assays screened with the 64-well Gene-Z card also showed some agreement between culture-based susceptibility tests and presence/absence of AR genes. Concerning only the antibiotics tested in which related AR genes were targeted on the Gene-Z card, 69 % (18/26) of culture-based resistance events were positive for an associated AR gene (Table S6). Resistance within the remaining eight events may be conveyed through other genes or mechanisms not targeted. In addition, a negative amplification event resulted in 100 % (15/15) correspondence with susceptibility.

For example, the *norA* gene encodes membrane-associated multidrug efflux pump and conveys resistance to (fluoro)quinolones (Tanaka et al. 2000). From tested antibiotics (Table 2), ciprofloxacin (Cip), gatifloxacin (Gat), and levofloxacin (Lev) belong to the fluoroquinolone drug class. Out of 11 tested isolates, nine were reported as fluoroquinolone resistant, eight isolates were resistant to all three tested antibiotics, and AR133 was solely resistant to ciprofloxacin. The *norA* gene amplified in eight isolates with multiple fluoroquinolone resistance (Cip + Gat + Lev). In isolate AR133, *norA* was not detected. However, ciprofloxacin resistance can be mediated through different mechanisms (Campion et al. 2004; Tanaka et al. 2000), and therefore, it can be hypothesized that resistance displayed by isolate AR133 was not due to the presence of *norA* gene. As described above, isolate AR133 is not *S. aureus* but rather a member of *S. intermedius*/*S. pseudintermedius* group, which is not listed to harbor the *norA* gene in the ARDB (Liu and Pop 2009).

Correspondence between the presence of *tetM* and *mecA* genes as measured with the 64-well Gene-Z card and resistance to associated antibiotics was also observed. The presence of the *tetM* gene was confirmed in two out of three tetracycline-resistant isolates. Tetracycline resistance can be conveyed by a number of genes. However, a majority of *S. aureus* strains carry either the *tetK* or *tetM* gene (Liu and Pop 2009). The *tetM* gene encodes a protein conferring resistance to tetracycline by interacting with the ribosome and promoting the release of bound tetracycline (Ito et al. 2003). Isolate AR137 may only harbor the *tetK* gene instead of the *tetM* gene. The presence of the *mecA* gene was confirmed in eight of the ten isolates that showed resistance to associated antibiotics. The *mecA* gene encodes penicillin binding protein (PBP2) and is accordingly involved in beta-lactam and consequently in methicillin resistance (Vannuffel et al. 1995). Even though, methicillin susceptibility was not tested, data was available for a range of other beta-lactam antibiotics including ampicillin (Amp), penicillin (Pen), and oxacillin (Oxa) (Hamilton et al. 2012). Ten out of 11 tested isolates showed beta-lactam resistance, nine isolates were resistant to all three tested antibiotics, and AR133 was solely resistant to penicillin. The *mecA* gene was observed absent for isolates AR131, AR133, and AR137. Isolate AR137 is susceptible to all tested beta-lactams, and therefore, this result is expected (true negative). In isolates AR131 and AR133, beta-lactam resistance may be conveyed through other genes or mechanisms.

The presence of the *ble* gene was observed in 6 out of the 11 isolates tested with the Gene-Z card. Since bleomycine resistance was not tested (Hamilton et al. 2012), results of LAMP assays could not be compared to phenotypic profiles. The *bl*e gene encodes binding protein with a strong affinity to the bleomycin family of antibiotics (Gennimata et al. 1996), which was included due to high incidence in screening initial isolates (9/10 *S. aureus* isolates).

Overall, screening results demonstrate the 64-well and 384-well Gene-Z cards and LAMP amplification are simple and rapid methods for multiple target isolate identification and screening of AR genes. The main benefit of this protocol is 30 min time to results. Including heat lysis step, reagent preparation, loading and sealing the card, and the typical ~20-min reaction time, the assay can be performed in less than 30 min. Results (Table S3) indicate non-lysed *S. aureus* cell template can be used directly with LAMP; thus in some instances, lysis may be unnecessary. If lysis is omitted and reagents are premixed, the assay can potentially be performed in under 25 min. PCR-based assays that require sample processing will necessitate more time and in some instances testing in an off-site diagnostics laboratory.

Additional advantages of LAMP integrated with the Gene-Z cards include (i) cost benefit, (ii) multiple assays in parallel, and (iii) size. Overall cost per sample is estimated to be $13 USD when using 384-well card, corresponding to 10 cents per test (with triplicate replications), with ~15 % of this cost from card material and fabrication and remaining percentage associated with reagent costs. Costs outside of an academic laboratory will increase for good manufacturing practices and quality control.

The footprint of the device and simplicity of minimal sample processing with LAMP offers the possibility of point of care, while the microfluidic self-dispensing Gene-Z cards can screen for multiple organism and AR genes in parallel. Development of a more comprehensive set of assays that focus on specific infections (e.g., blood- or urine-based assays) will add utility to the Gene-Z device for LAMP diagnostics in a clinical setting. For example, a user could design cartridges with assays to identify the 20 most common organisms responsible for ~90 % of septic infections and also target AR genes associated with resistance in a high percentage of those organisms. It should be acknowledged that molecular-based assays could fail to identify an infectious organism due to exclusion or incomplete coverage of primers, or instances in which the presence or absence of an AR gene does not correspond with susceptibility.

Taken together, DNA-based molecular detection of AR genes cannot replace culture-based susceptibility tests. The molecular presence and absence of AR genes can only offer inferences towards a potential means or confirmation of treatment. This is particularly useful when DNA-based means of detection are simple, rapid, and offer testing for difficult-to-culture microorganisms such as *Mycobacterium* and *Chlamydia*. A RNA-based approach with a brief pulse of antibiotics followed by the identification of transcripts (Barczak et al. 2012) using RT-LAMP (Rudolph et al. 2015) and the Gene-Z card or similar devices may provide molecular results equivalent to susceptibility testing.

## Electronic supplementary material

ESM 1 (PDF 734 kb)
