# Supplementary material for: Thirty-minute screening of antibiotic resistance genes in bacterial isolates with minimal sample preparation in static self-dispensing 64 and 384 assay cards
Source: Appl Microbiol Biotechnol. 2015 Jul 31;99(18):7711–22. doi: 10.1007/s00253-015-6774-z (PMC4543423; doi:10.1007/s00253-015-6774-z)
Supplement: Supplementary file 1 — (PDF 734 kb) [file 253_2015_6774_MOESM1_ESM.pdf]

Supplemental File

Applied Microbiology and Biotechnology

30 min screening of antibiotic resistance genes in bacterial isolates with minimal sample-preparation in static self-dispensing 64 and 384 assay cards

Tanja Kostić[1], Michael Ellis[2,*], Maggie Kronlein[2], Tiffany M. Stedtfeld[2], John B. Kaneene[3], Robert D. Stedtfeld[2], and Syed A. Hashsham[2,#]

[1]AIT Austrian Institute of Technology GmbH, Bioresources Unit, Konrad Lorenz Strasse 24, A-3430 Tulln an der Donau, Austria

[2] Michigan State University, Civil and Environmental Engineering, East Lansing, MI, 48824, USA

[3] Michigan State University, Center for Comparative Epidemiology, College of Veterinary Medicine, East Lansing, MI, 48824, USA

[*]currently at Barr Engineering Company, Ann Arbor, MI 48108

[#]corresponding author: Dr. Syed A. Hashsham

Michigan State University

Civil and Environmental Engineering

East Lansing, MI, 48824

hashsham@egr.msu.edu

Supplemental File includes – Pictures of Gene-Z chip captured during 60 min reaction (Fig S1) amplification curves for gDNA, lysed and native cell templates (Fig S2), a list of all primers used in this study (Table S1), a list of antibiotic resistance genes in *E. faecalis*, *E. faecium* and *S. aureus* (Table S2), results of sample preparation experiment (Table S3), screening results for *E. faecalis, E. faecium* and *S. aureus* (Table S4*)*.

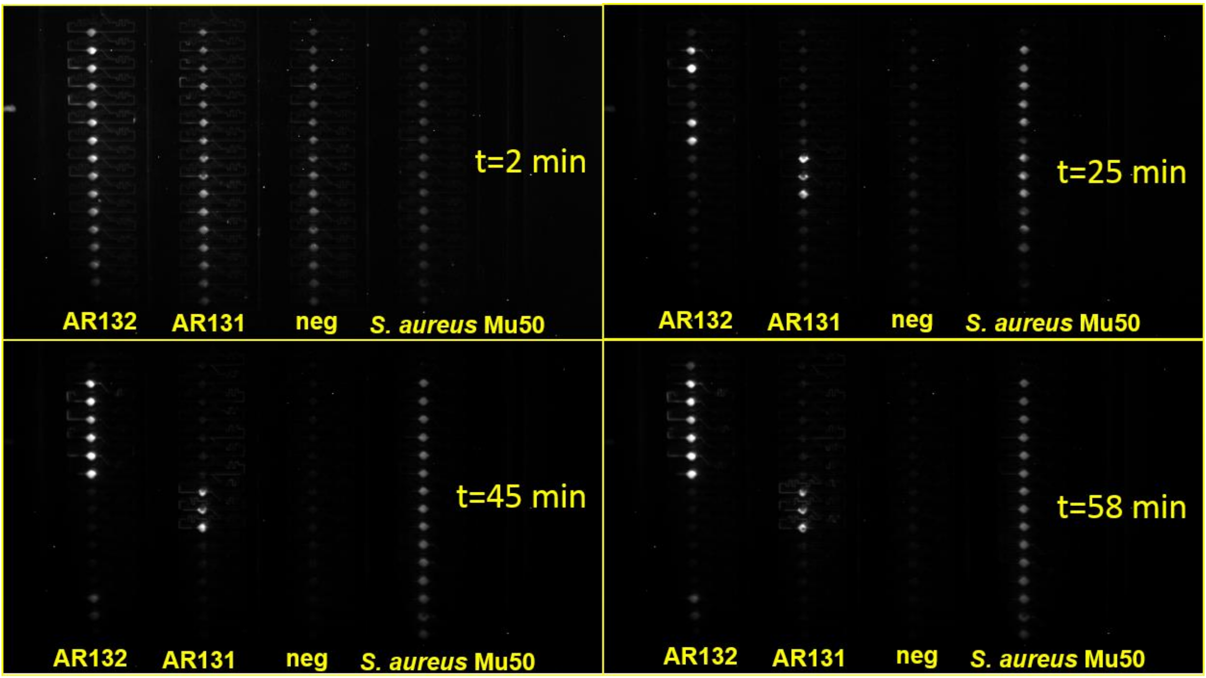

Fig S1. Images of Gene-Z chip captured during 60 min reaction to demonstrate background observed in initial images. LAMP experiments was performed with crude heat lysed non purified cell templates.

A)

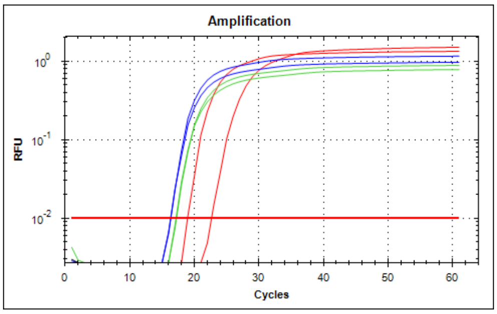

B)

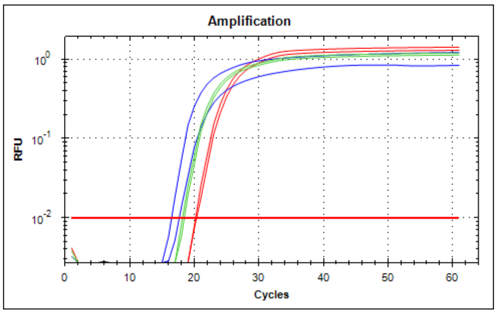

C)

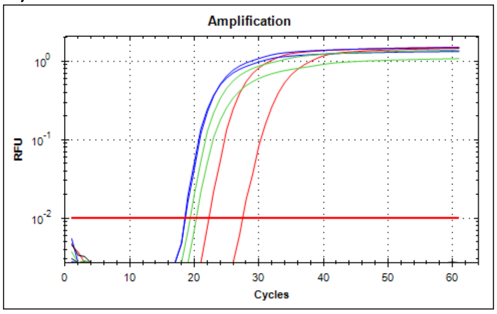

Fig S2. Amplification curves for gDNA (red), heal lysed non purified crude cells (blue) and native (green) cell templates of *S. aureus* isolate AR132. Experiments tested at isothermal conditions in thermal cycler (Chromo4^{TM}, Biorad). a) *nuc* b) *mecA* and c) *norA* assay . Negative control is in black. Baseline set at 0.01 RFU. One amplification cycle = 45 seconds. Negative controls were included, but not visible in these graphs. Experiments with cells and gDNA were performed with $5x10^5$ cfu/reaction and 1 ng (approx. 3.3x 10^5 genome copies)/reaction, respectively.

Table S1. List of LAMP primers.

primer set		primer sequence (5' - 3')	reference strain
ant3ia	F3	GAGATTCTCCGCGCTGTAG	*A. baumanii* AYE (ATCC BAA-1710)
	B3	CAAAGAGTTCCTCCGCCG	
	FIP	GCAGTTCGCGCTTAGCTGGA-TCACCATTGTTGTGCACGAC	
	BIP	CAGGTATCTTCGAGCCAGCCAC-CCAAGGCAACGCTATGTTCT	
	LB	AACGCCACGGAATGATGTC	
	LF	CATTGATCTGGCTATCTTGCTGACA	
aadD	F3	ATGATGTTAAGGCTATTGGTG	*S. aureus* Mu50 (ATCC 700699)
	B3	GCCAATCTGATTCCACCT	
	FIP	TGACATGACACACATCATCTCAATA-TTTATGGCTCTCTTGGTCG	
	BIP	ARGAAGCAGAGTTCAGCCATGA-TCTAGTAGAATCTCTTCGCTATC	
	LB	GAATAGGGCCCATCAGTCTGA	
	LF	ATGGACAACCGGTGAGTGG	
bacA	F3	GAATTTTTAGGTTCTCAATCAGC	*S. aureus* Mu50 (ATCC 700699)
	B3	TGTTCTTCGATGAAATCATCAA	
	FIP	AATCTCTAAGAAGCGTTCGCG-CATCCAATTAGGATCCGTCT	
	BIP	ATGTTGAAGGAGAWAACRATCAACA-AGCCTAAAATACCTGCTGG	
	LB	GAATACCCATGCTGCTGCAA	
	LF	AAGACGTTCAAAGCCAAGACG	
bl2b_tem	F3	TTTGCACAACATGGGGGAT	*E. fergusonii* (ATCC 35469)
	B3	TGGTCCTGCAACTTTATCCG	
	FIP	TGTCACGCTCGTCGTTTGGTAT-TAACTCGCCTTGATCGTTGG	
	BIP	CCACGATGCCTGTAGCAATGGC-TGTTGCCGGGAAGCTAGA	
	LB	GCTTCATTCAGCTCCGGTTC	
	LF	ACAACGTTGCGCAAACTATTAACTG	
ble	F3	GTACCAGCAATAAACGACTC	*S. aureus* Mu50 (ATCC 700699)
	B3	AGTCGGGGATATTAAAAAGAGT	
	FIP	CGGATTCATCTATGGGAGGCA-CCGGTGAATCATTACTACGA	
	BIP	AACCTCATTACACATTAGAACTGCG-<br>GGTTTTTATTGCGATAAACTAGG	
	LB	GTGATGAAGGCTGGCGC	
	LF	AATCCATCTTCATGGTGAACCAAAG	
cata9	F3	CGACTTTTAGTATAACCACAGAA	*S. pneumoniae* (ATCC 700669)
	B3	TTACAGGAGTCCAAATACCA	
	FIP	TGAGTTTATCACCCTTGTCACTAAG-<br>CCGAAACATAAAACAAGAAGGA	
	BIP	GTTACAATAGCGACGGAGAGTTAGG-<br>GAGAATGTTTTAGATACACCATCAA	
	LB	GGGATAAGTTAGAGCCACTTTATACA	
	LF	n.a.	
dfra12	F3	CGCAGACTCACTGAGGGAA	*n.a.*
	B3	ATTGCCGAGTTCGGATGC	
	FIP	ACCAATGTGTGACGGTTCGGT-GTCATGGGGCGAAAGACC	
	BIP	ACGCCAAGCTAACTACCGCG-AAAGCGATAGCGTGCGAC	
	LB	AGGCTTGCCGATAGACTCAAA	
	LF	CCACTGGCTGCGTAGTTGT	
lsa	F3	TTTGCCTATGATAATCAAGAAGY	*E. faecalis* V583 (ATCC 700802)

	B3	GTTCAAAAGAAGTCACCTCTT	
	FIP	TAAGGTKGTTTTCCCACGGCC-TTGATCAGGCAAATATCACG	
	BIP	ACCAAGGAGAGATTCTTCATCAAG-CATAATAAGTSAGCTGTTGTTCT	
	LB	CCAATCAATCCTAATTTCCAATTGGTATCC	
	LF	ATTTCGTCTATTTTCCACAAACAGTTGCRG	
mphC	F3	GACTGAAGCAACCCACTC	*n.a.*
	B3	CGTTTCATACGCCGATTC	
	FIP	CCRTATGCTGTTATRAGTTGCTCTA-TCAATGGACTTTATAGGACACC	
	BIP	AYATGGCCACGAATGAAAGARC-CTGATTCCATAGCAAACTCAG	
	LB	CCTTCGTCGTCGAATACACGAT	
	LF	AGAACTCAATGCAGTATTCCCAATG	
mepA	F3	GGACATCATATGGTCGGAC	*S. aureus* Mu50 (ATCC 700699)
	B3	GCAATTAATAATGACCAAATGACAC	
	FIP	AGCATACCAGTAAACAAGAAACCTA-TACTGATCAAGCCATTGTTGA	
	BIP	GCAAGGTCGTGGTGCTACAA-GTTAGTCCAAACAAAGCATTC	
	LB	GCCATTGTTACTTTCAAAATAAATGTCGCC	
	LF	TTATGGCCATTTTACAAGGTGCA	
norA	F3	TTGGTGGTATGAGTGCTG	*S. aureus* Mu50 (ATCC 700699)
	B3	CCTAATGCTCCTGCAAAGT	
	FIP	CCAAAGTTTTTTGCTTTTTGATGGC-GTATGGTAATGCCTGGTGTG	
	BIP	TTCWGGATTCATTTTAGGACCAGG-AAAATGGCATACGATGTGAA	
	LB	GGTGAAATGTCAGCTATTAAACCTG	
	LF	GATTGGTGGATTTATGGCAGAAGT	
qacA	F3	CCATTATCATATTCTACAATGGCT	*S. aureus* Mu50 (ATCC 700699)
	B3	TCTCCAACTATACCTTTTGATGA	
	FIP	CCTGCTTTTGATGTAGGTGTTTCTA-CATTAATTTTAGTTGGAGCTGGT	
	BIP	TGTATGACCTTGGAAATGTTTTTGG-AATACACGATAAAGCATAGAAGA	
	LB	TGTAGCAGTACTTGGTAGCCT	
	LF	n.a.	
tetO	F3	ACAGAGATGTGTGTTCCG	*S. pyogenes* (ATCC BAA-1064)
	B3	ATTTCCCGCTGTTCAGAT	
	FIP	GTTTAGCTGCAAAACATCATTTGGT-ACAAACGGTGAATTATATTCATCC	
	BIP	CTGTTGCCGCAGAGAAAATTTATT-TTCTTTACTGCAATCGTTGTT	
	LB	TCACCAGAGCAGGCTGTATC	
	LF	GAAAATCCTCTCCCTATGCTCCA	
tetM	F3	CTTATTATATAACAGTGGAGCGATT	*S. aureus* Mu50 (ATCC 700699)
	B3	AATTGCCCCATCTAAAACTG	
	FIP	CCTCTCTGACGTTCTAAAAGCGTAT-AGAATTAGGAAGCGTGGAC	
	BIP	ACCTCTTTTCAGTGGGAAAATACGA-ATGAACGATATACTTCTGCTAAG	
	LB	TCCGTCCTCGTTGTACCTTT	
	LF	AGGTGAACATCATAGACACGCCAG	
vanG	F3	GCTCTGATTTCCGCATCT	*n.a.*
	B3	CAAGGCATTAATACCTCTGTT	
	FIP	CCACCAAAGCCTTTTCATAATCTTT-CTTAGTTTATTAAGCGGGCAT	

	BIP	GTGTTTCGGGACAATAAAGAATGGT-TGAAGCCTTTCAGATACAWYS	
	LB	CCCCACAACAGCCCTTGAAA	
	LF	TTGTGATGATAGCTGCATTTGTSK	
vanB	F3	GTTCCTCGACCGGAATGTC	*E. faecalis* V583 (ATCC 700802)
	B3	GCRGCAGGACAATATGATGG	
	FIP	CCACGGTATCTTCCGCATCCAT-TGCGGGAACTGTAATCATCG	
	BIP	CGGATTTGATCCACTTCGCCGA-CGATTTCGGGCTGTGAGG	
	LB	GCCGGAAAAAGGCTCAGAAAATG	
	LF	CAATCAAATCATCCTCGTTYCCC	
vanA	F3	TTGCCGTTTCCTGTATCCG	*n.a.*
	B3	CTGTGAGGTCGGTTGTGC	
	FIP	CGAGCCGGAAAAAGGCTCTGAA-CCTCGCTCCTCTGCTGAA	
	BIP	TCCGTACTGCAGCCTGATTTGG-TATTGGGAAACAGTGCCGC	
	LB	GTTATAACCGTTCCCGCAGAC	
	LF	TCCACCTCGCCAACAACTA	
vanYA	F3	TGATGAGCAAAGTGTGCT	*n.a.*
	B3	TTGAATTCCTGTTAACTCTGTT	
	FIP	GTCAAGCTTGATCCTACATCTAGTG-AAGAAATGGGGGCTGAGT	
	BIP	AACGAGCCCCTGAAGGAAAG-TTGTCCTCTGGATAACGTAA	
	LB	ACTATAACCTGCTGGTAAGGCAT	
	LF	TGCTTGGAAATACGGGTTCATT	
vatA	F3	GGATGGGTTGGGAGAAGT	*n.a.*
	B3	CGATAACTCCATCAGAAAACCT	
	FIP	CAGGCATAATGGTTACATCTCTACC-CTTCCTTAAAAGATCTTCCCTTG	
	BIP	GGCAATCATTGCTGCAGAAGC-GGGATTTCCACCGACAAT	
	LB	CATCATTTCCAATTTCAATGTCCCC	
	LF	TTGTCACAAAGAATGTTGCTCCCTA	
vgbA	F3	CACTTTTGATTTGAATTGGGTAT	*n.a.*
	B3	GGTTTGTGGAAATTATCGGTAA	
	FIP	TGGAGCAGGAATTGATTTATGGTTT-CCTCAATTATATTATTGCTTGTCAG	
	BIP	GCAGTAATTGCATGAGGTCGAG-AGATAGGGCGAATAACTCCT	
	LB	GGGGGGCTAATAAAATAGGAAGG	
	LF	CGTTTGGCGTTGGAATTTTGAA	

n.a. - none available

additional primer set used in 2nd screening

**primer set**		**primer sequence (5' - 3')**
nucA	F3	AACAGTATATAGTGCAACTTCAA
	B3	CTTTGTCAAACTCGACTTCAA
	FIP	ATGTCATTGGTTGACCTTTGTACAT-AAATTACATAAAGAACCTGCGA
	BIP	TATTGGTKGATACACCTGAAACAAA-ATTTTTTTCGTAAATGCACTTGC
	LB	AGGTGTAGAGAAATATGGTCCTGAA
	LF	ATTTAACCGTATCACCATCAATCGC
mecA	F3	ATCTCATATGCTGTTCCTGTA

	B3	AAAAAACGAGTAGATGCTCAA
	FIP	AATGCAGAAAGACCAAAGCATACATGCCAATTCCACATTGTTTCG
	BIP	TGACGCTATGATCCCAATCTAACTACTACGGTAACATTGATCGC
	LB	CCACATACCATCTTCTTTAACAAAATTAAATTG
	LF	TTTAAAATCAGAACGTGGTAAAATTTTAGAC
Staph_16S	F3	GGTCCCCGTCAATTCCTTTG
	B3	GAACACCAGTGGCGAAGG
	FIP	AGGGGGTTTCCGCCCCTTAGAGTTTCAACCTTGCGGTCG
	BIP	CTCATCGTTTACGGCGTGGACTCTGACGCTGATGTGCGAA
	LB	TAAGCACTCCGCCTGGG
	LF	CCAGGGTATCTAATCCTGTTTGATC

Table S2. MIC values for the *S. aureus* isolates listed in table 2. Abbreviations: Amp – Ampicillin, Pen – Penicillin, Rif – Rifampin, Cli – Clindamycin, Ery – Erythromycin, Oxa – Oxacillin, Syn – Quinupristin-Dalfopristin, Tet – Tetracycline, Cip – Ciprofloxacin, Gat – Gatifloxacin, Lev – Levofloxacin, Cef – Ceftriaxone, Tri – Trimethoprim-Sulfamethoxazole, Van – Vancomycin, Gen – Gentamicin.

Antibiotic/Iso.	AR131	AR132	AR133	AR134	AR135	AR136	AR137	AR139	AR141	AR142	AR143
AMP	1	8	<0.1	>16	8	>16	<0.1	>16	>16	>16	>16
CEF	<8	16	<8	>64	>64	>64	8	>64	>64	32	>64
CIP	<0.5	>2	>2	>2	>2	>2	>2	2	>2	>2	>2
CLIN	>2	<0.1	>2	>2	<0.1	>2	<0.1	>2	>2	<0.12	>2
ERY	>4	>4	>4	>4	>4	>4	>4	>4	>4	>4	>4
GAT	<1	4	<1	8	4	8	>8	<1	>8	8	8
GEN	8	<2	<2	<2	<2	<2	2	16	<2	<2	<2
LEV	<0.2	8	2	>8	>8	>8	>8	1	>8	>8	>8
OXA	>8	>8	<0.2	>8	>8	>8	0.2	>8	>8	>8	>8
PEN	4	>8	0.5	>8	>8	>8	0.06	>8	>8	>8	>8
SYN	>4	0.25	0.25	0.5	0.25	0.5	>4	1	0.5	0.25	1
RIF	>4	<0.5	<0.5	<0.5	<0.5	<0.5	>4	<0.5	<0.5	<0.5	<0.5
TET	>16	<2	>16	<2	<2	<2	>16	<2	<2	<2	<2
TRI	<0.5	<0.5	<0.5	<0.5	<0.5	<0.5	>4	<0.5	<0.5	<0.5	<0.5
VAN	<1	<1	<1	<1	<1	<1	>128	2	<1	<1	<1

Table S3. List of all antibiotic resistance genes listed for *E. faecalis, E. faecium* and *S. aureus* (ARDB; June, 2010).

Organism	Antibiotic Resistance Genes
E. faecalis	*aad9, ant3ia, bl2be_shv2, emeA,lsa, tetO, tetT, vanE, vanG, vanRE, vanRG, vanES, vanSG, vanTE, vanTG, vanUG, vanWG, vanXYE, vanXYG, vanYD, vanYG*
E. faecium	*tetU, vanD, vanHD, vanRD, vanXD, vatD, vatE, vanSD*
S. aureus	*aacA4, aac6ib, aadA2, aadA5, aadD, bl2B_tem, ble, cml_e1, cml_e4, cmlA1, dfra17, ermA, ermC, ermGM, ermY, fusB, linA, lnuA, mecr1, mepA, mphC, msrA, norA, qac, qacA, qacB, tet38, tetK, vatA, vatB, vgaA, vgaB, vgbA*
E. faecalis , E. faecium	*aph2, lnub, msrC, pbp, tetS, vanB, vanHB,, vanRB, vanSB, vanWB, vanXB, vanYB*
E. faecalis, S. aureus	*bacA, bl2a_pc, cata7, dfra12, mecA*
E. faecium, S. aureus	*cata8, cata9, mefA, str*
E. faecalis , E. faecium, S. aureus	*aac6ie, aad9ib, aph3iiia, ermB, tetL, tetM, vanA, vanHA, vanRA, vanSA, vanXA, vanYA, vanZ*

Table S4. Results of threshold time from sample preparation experiment (n.d. – not detected) tested with gDNA, crude heat lysed, and native cells of *S. aureus* isolate AR132. Mean threshold time (Tt in min) and standard deviation from two replicates.

	**gDNA (1 ng ~ 3.3x10^5 genomic copies)**		**Lysate (5x10^5 cfu)**		**Cells (5x10^5 cfu)**		**Negative control**
	**Tt mean**	**Tt stdev**	**Tt mean**	**Tt stdev**	**Tt mean**	**Tt stdev**	
nuc	15.5	1.92	12.2	0.02	12.8	0.03	n.d.
mecA	15.1	0.07	12.7	0.64	13.7	0.11	n.d.
norA	18.6	2.76	13.8	0.05	14.8	0.51	n.d.
ble	n.d.		n.d.		n.d.		n.d.
tetM	n.d.		n.d.		n.d.		n.d.

Table S5. Screening results in terms of presence (+) and absence (-) with the first group of 30 *E. faecalis*, *E. faecium* and *S. aureus* isolates. Assays were tested under isothermal conditions using the Chromo4 (Biorad). Isolate numbers are internally designated.

		**Amplification result**									
**Species**	**Isolate Number**	***ant3ia***	***bacA***	***dfra12***	***lsa***	***tetO***	***tetM***	***vanG***	***vanB***	***vanA***	***vanYA***
E. faecalis	AR 101	-	-	-	-	-	-	-	-	-	-
	AR 102	-	-	-	-	-	-	-	-	+	-
	AR 103	-	-	-	-	-	+	-	-	-	-
	AR 104	-	-	-	-	-	+	-	-	-	-
	AR 105	-	-	-	-	-	-	-	-	-	-
	AR 106	-	-	-	-	-	-	-	-	+	+
	AR 108	-	+	-	-	-	+	-	-	+	+
	AR 109	-	-	-	-	-	-	-	-	+	-
	AR 110	-	-	-	-	-	+	-	-	+	-
	AR 111	-	-	-	-	-	-	-	-	+	-

**Species**	**Isolate Number**	***cata9***	***tetM***	***vanB***	***vanA***	***vanYA***
E. faecium	AR 107	-	+	-	+	+
	AR 112	-	+	-	+	+
	AR 113	-	+	-	+	+
	AR 114	-	+	-	+	+
	AR 115	-	+	-	+	+
	AR 116	-	-	-	-	-
	AR 117	-	-	-	-	-
	AR 118	-	-	-	-	-
	AR 119	-	-	-	-	-
	AR 120	-	+	-	-	-

**Species**	**Isolate Number**	***aadD***	***bacA***	***bl2B tem***	***ble***	***cata9***	***dfra12***	***mphC***	***mepA***	***norA***	***qacA***	***tetM***	***vanA***	***vanYA***	***vatA***	***vgbA***
S. aureus	AR 121	-	+	+	+	-	-	+	+	+	-	-	-	-	+	-
	AR 122	-	+	+	+	-	-	-	+	+	-	-	-	-	-	-
	AR 123	-	+	+	+	-	-	-	-	+	-	+	-	-	-	-
	AR 124	-	+	+	+	-	-	-	+	+	-	+	-	-	-	-
	AR 125	-	+	+	+	-	-	+	+	+	-	+	-	-	-	-
	AR 126	-	+	+	+	-	-	-	-	+	-	+	-	-	-	-
	AR 127	-	+	+	+	-	-	-	-	+	-	+	-	-	-	-
	AR 128	-	+	+	+	-	-	-	-	+	-	+	-	-	-	-
	AR 129	-	+	+	-	-	-	+	+	+	-	-	-	-	-	-
	AR 130	-	+	+	+	-	-	+	+	+	-	-	+	-	-	-

Table S6. Correspondence of culture based susceptibility and AR genes detected with LAMP on Gene-Z card (using crude heat lysed non purified cell template). This table only includes the antibiotics used in culture based susceptibility tests related to targeted AR genes included in the LAMP assay. Abbreviations: Amp – Ampicillin, Pen – Penicillin, Ery – Erythromycin, Oxa – Oxacillin, Tet – Tetracycline, Cip – Ciprofloxacin, Gat – Gatifloxacin, Lev – Levofloxacin, Tri – Trimethoprim-Sulfamethoxazole.

AR culture / isolates tested	AR gene detected / AR culture	True negative
Tri (1/11)	drA12 (0/0)	(4/4)
Ery (11/11)	mphC (1/4)	-
Cip, Gat, Lev (9/11)	norA (8/9)	(2/2)
Tet (3/11)	tetM (2/3)	(8/8)
Amp, Pen, Oxa (10/11)	mecA (7/10)	(1/1)
Total	18/26	15/15
